# Supplementary material for: HIVprotI: an integrated web based platform for prediction and design of HIV proteins inhibitors
Source: J Cheminform. 2018 Mar 9;10:12. doi: 10.1186/s13321-018-0266-y (PMC5845081; doi:10.1186/s13321-018-0266-y)
Supplement: Supplementary file 2 — Additional file 2. Source code of HIVProtI web server. [file 13321_2018_266_MOESM2_ESM.zip › HIVprotI_Source-code/hivprotI/weka-3-6-6/doc/weka/associations/tertius/IndividualInstances.html]

IndividualInstances


---


|  |  |  |
| --- | --- | --- |
| **Overview** \| **Package** \| **Class** \| **Tree** \| **Deprecated** \| **Index** \| **Help** \| **Weka's home** | |  |
| **PREV CLASS**   **NEXT CLASS** | **FRAMES**    **NO FRAMES**     **All Classes** |  |
| SUMMARY: NESTED \| FIELD \| CONSTR \| METHOD | DETAIL: FIELD \| CONSTR \| METHOD |  |


---


## weka.associations.tertius Class IndividualInstances

```
java.lang.Object
  weka.core.Instances
      weka.associations.tertius.IndividualInstances
```

**All Implemented Interfaces:**: java.io.Serializable, RevisionHandler

---

``` public class IndividualInstances extends Instances ```

**Version:**
:   $Revision: 1.6 $

**Author:**
:   Peter A. Flach, Nicolas Lachiche

**See Also:**: Serialized Form

---

**Field Summary**

**Fields inherited from class weka.core.Instances**

`ARFF_DATA, ARFF_RELATION, FILE_EXTENSION, SERIALIZED_OBJ_FILE_EXTENSION`


**Constructor Summary**

`IndividualInstances(Instances individuals,
Instances parts)`


| **Method Summary** | |
| --- | --- |
| `java.lang.String` | `getRevision()`             Returns the revision string. |

**Methods inherited from class weka.core.Instances**

`add, attribute, attribute, attributeStats, attributeToDoubleArray, checkForAttributeType, checkForStringAttributes, checkInstance, classAttribute, classIndex, compactify, delete, delete, deleteAttributeAt, deleteAttributeType, deleteStringAttributes, deleteWithMissing, deleteWithMissing, deleteWithMissingClass, enumerateAttributes, enumerateInstances, equalHeaders, firstInstance, getRandomNumberGenerator, insertAttributeAt, instance, kthSmallestValue, kthSmallestValue, lastInstance, main, meanOrMode, meanOrMode, mergeInstances, numAttributes, numClasses, numDistinctValues, numDistinctValues, numInstances, randomize, readInstance, relationName, renameAttribute, renameAttribute, renameAttributeValue, renameAttributeValue, resample, resampleWithWeights, resampleWithWeights, setClass, setClassIndex, setRelationName, sort, sort, stratify, stringFreeStructure, sumOfWeights, swap, test, testCV, toString, toSummaryString, trainCV, trainCV, variance, variance`

**Methods inherited from class java.lang.Object**

`equals, getClass, hashCode, notify, notifyAll, wait, wait, wait`

**Constructor Detail**

### IndividualInstances

```
public IndividualInstances(Instances individuals,
                           Instances parts)
                    throws java.lang.Exception
```

**Throws:**: `java.lang.Exception`


**Method Detail**

### getRevision

```
public java.lang.String getRevision()
```

:   Returns the revision string.

    :   **Specified by:**: `getRevision` in interface `RevisionHandler` **Overrides:**: `getRevision` in class `Instances`
    :   **Returns:**: the revision


---


|  |  |  |
| --- | --- | --- |
| **Overview** \| **Package** \| **Class** \| **Tree** \| **Deprecated** \| **Index** \| **Help** \| **Weka's home** | |  |
| **PREV CLASS**   **NEXT CLASS** | **FRAMES**    **NO FRAMES**     **All Classes** |  |
| SUMMARY: NESTED \| FIELD \| CONSTR \| METHOD | DETAIL: FIELD \| CONSTR \| METHOD |  |


---
